# Supplementary material for: Lamivudine (3TC), a Nucleoside Reverse Transcriptase Inhibitor, Prevents the Neuropathological Alterations Present in Mutant Tau Transgenic Mice
Source: Int J Mol Sci. 2023 Jul 6;24(13):11144. doi: 10.3390/ijms241311144 (PMC10342792; doi:10.3390/ijms241311144)
Supplement: Supplementary file 1 [file ijms-24-11144-s001.zip › ijms-2476408-Supplementary.pdf]

Supplementary Figures

Lamivudine (3TC), a Nucleoside Reverse Transcriptase Inhibitor, Prevents the Neuropathological Alterations Present in Mutant Tau Transgenic Mice

Laura Vallés-Saiz, Jesús Ávila and Félix Hernández *

Centro de Biología Molecular "Severo Ochoa", CSIC/UAM, Universidad Autónoma de Madrid, Cantoblanco, 28049 Madrid, Spain; laura.valles@cbm.csic.es (L.V.-S.); javila@cbm.csic.es (J.Á.)

* Correspondence: felix.hernandez@uam.es; Tel.: +34-91-196-45-63; Fax: +34-91-196-44-20

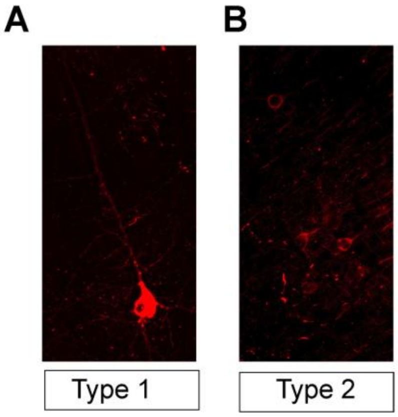

Supplementary Figure S1. Representative AT8+ Type 1 or "Tangle-like" neuron (A) and Type 2 neuron with a diffuse pattern (B).

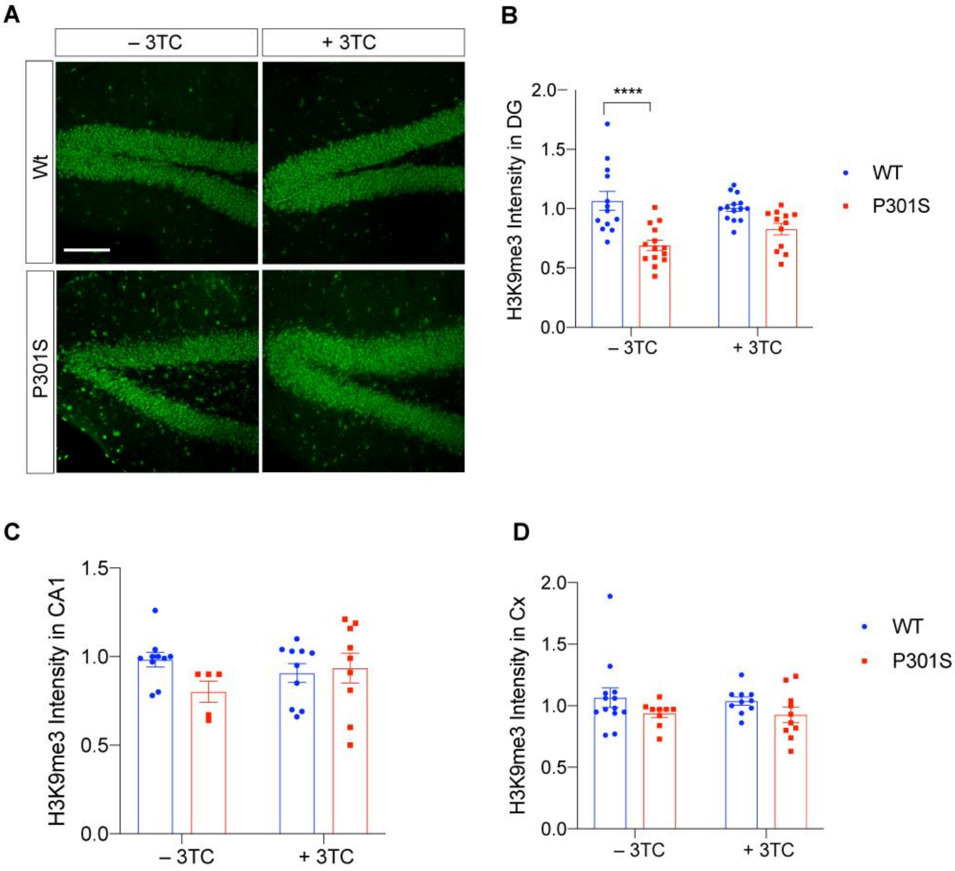

Supplementary Figure S2. 3TC treatment promotes compaction of heterochromatin in P301S mice. (A) Representative images of the dentate gyrus with the H3K9me3 marker of slices from mice treated with 3TC and untreated counterparts and their quantification. (B) The graph shows the mean± SEM (n= 13–15 per condition). ****p<0.0001 using two-way ANOVA followed by Student's t-test for comparisons. Scale bar, 20 µm. (C-D) 3TC treatment did not promote the compaction of heterochromatin in the CA1 and cortex of wild-type or P301S mice with the H3K9me3 marker. Graphs show the mean± SEM (n= 13–15 per condition). Two-way ANOVA did not show statistically significant differences between the means. Scale bar, 20 µm.
